# The coordinated roles of miR-26a and miR-30c in regulating TGFβ1-induced epithelial-to-mesenchymal transition in diabetic nephropathy

**DOI:** 10.1038/srep37492

**Published:** 2016-11-22

**Authors:** Zongji Zheng, Meiping Guan, Yijie Jia, Dan Wang, Ruoyu Pang, Fuping Lv, Zhizhou Xiao, Ling Wang, Hongbin Zhang, Yaoming Xue

**Affiliations:** 1 Department of Endocrinology and Metabolism, Nanfang Hospital, Southern Medical University, Guangzhou, 510515, China; 2 Department of Biomedical Sciences, University of Copenhagen, Copenhagen, 2200, Denmark

## Abstract

MicroRNAs (miRNAs) play vital roles in the development of diabetic nephropathy. Here, we compared the protective efficacies of miR-26a and miR-30c in renal tubular epithelial cells (NRK-52E) and determined whether they demonstrated additive effects in the attenuation of renal fibrosis. TGFβ1 suppressed miR-26a and miR-30c expression but up-regulated pro-fibrotic markers in NRK-52E cells, and these changes were also found in the kidney cortex of 40-week-old diabetic Otsuka Long-Evans Tokushima fatty (OLETF) rats. Bioinformatic analyses and luciferase assays further demonstrated that both miR-26a and miR-30c targeted connective tissue growth factor (CTGF); additionally, Snail family zinc finger 1 (Snail1), a potent epithelial-to-mesenchymal transition (EMT) inducer, was targeted by miR-30c. Overexpression of miR-26a and miR-30c coordinately decreased CTGF protein levels and subsequently ameliorated TGFβ1-induced EMT in NRK-52E cells. Co-silencing of miR-26a and miR-30c exhibited the opposite effect. Moreover, miR-26a and miR-30c co-silenced CTGF to decrease ERK1/2 and p38 MAPK activation. Furthermore, miR-26a was up-regulated in urinary extracellular vesicles of diabetic nephropathy patients. Our study provides evidence for the cooperative roles of miR-26a and miR-30c in the pathogenesis of diabetic nephropathy, and the co-targeting of miR-26a and miR-30c could provide a new direction for diabetic nephropathy treatment.

Diabetic nephropathy (DN) is commonly recognized as a leading cause of chronic kidney disease worldwide^1^. DN is characterized by excessive extracellular matrix (ECM) deposition in the renal tubulointerstitium and glomerulus, and this deposition can develop into interstitial fibrosis and glomerulosclerosis. The tubulointerstitium accounts for 90% of the volume of the kidney^2^, and tubulointerstitial fibrosis has been shown to be the best histological predictor of DN progression^3^. It is well known that myofibroblasts are important for ECM synthesis and secretion. Numerous studies have demonstrated that the epithelial-to-mesenchymal transition (EMT) contributes to matrix generation in kidney disease and tubular epithelial cells and that EMT is crucial for tubulointerstitial fibrosis^4^. Previous studies using diabetic animal models and kidney biopsies from DN patients have demonstrated that TGFβ1-induced EMT is responsible for tubulointerstitial fibrosis in DN^5^,^6^,^7^ and that EMT contributes to the generation of 36% of all myofibroblasts that are formed during kidney fibrosis^8^.

Connective tissue growth factor (CTGF) is one of the critical factors that regulates DN. CTGF belongs to the CCN protein family^9^, which plays key roles in regulating ECM synthesis^10^. In patients with type 1 diabetes mellitus (DM), a high plasma CTGF level is closely associated with mortality from end-stage renal disease associated with DN^11^, and urinary CTGF excretion is strongly linked to DN severity^12^. In addition, increased CTGF expression in biopsy specimens from patients at different stages of DN is correlated with the progression of DN^13^. Furthermore, patients with microalbuminuria who were treated with anti-CTGF monoclonal antibodies showed a decrease in albuminuria^14^.

miRNAs are endogenous, small, noncoding RNAs of ~22 nucleotides that play key roles in the posttranscriptional repression of target gene expression by binding to the 3′ untranslated regions (3′-UTRs) of mRNAs^15^. miRNAs are vital to the pathogenesis of multiple diseases, including DN, and they have become an intriguing target for therapeutic intervention. miR-23b^16^, miR-21^17^, miR-200^18^, miR-141^19^ and miR-130b^20^ have been reported to be involved in EMT in the kidney, which is involved in the pathogenesis of DN. In addition, we and others have shown that miRNAs might be valuable diagnostic markers because they are also present in urine^21^,^22^.

Previous studies have found that many miRNAs target CTGF and thus alleviate ECM synthesis. miR-26a directly targets the CTGF 3′-UTR in MRC-5 cells^23^ and targets both the CTGF and Col-I genes in cardiac fibroblasts^24^. miR-26a inhibits TGFβ1-induced ECM protein expression by targeting CTGF in podocytes^25^. miR-30c and miR-133 reduce CTGF expression in cardiac fibrosis^10^. Overexpression of miR-30c may ameliorate kidney fibrosis in DN by inhibiting CTGF expression^26^. In addition, miR-18a and miR-19b directly target CTGF in age-related cardiac remodeling^27^. miR-143 targets CTGF in hypertrophic scar fibroblasts^28^. However, the role of CTGF-related miRNAs in tubular epithelial cells has not been examined.

Recently, certain miRNAs were found to synergize in the regulation of pathophysiological processes in multiple organs. miR-34 and let-7 synergistically restrain tumor growth in non-small cell lung cancer^29^. miR-143 and miR-145 co-inhibit ERBB3 in breast cancer^30^, and miR-497 and miR-34a cooperatively target cyclin E1 in lung cancer^31^. Many studies have explored the role of single novel miRNAs in the pathogenesis of DN, but few have examined the synergistic effects of miRNAs in the regulation of EMT in DN. We hypothesized that key miRNAs may synergistically target individual critical genes to form a network that regulates DN. Based on previous studies and computational miRNA target site prediction algorithms, we hypothesized that miR-26a and miR-30c may co-target CTGF. Herein, we performed experiments to test our hypotheses that miR-26a and miR-30c coordinate the inhibition of CTGF expression and consequently suppress EMT in NRK-52E cells.

## Results

### Decreased miR-26a and miR-30c expression in TGFβ1-treated NRK-52E cells

**Figure 1:**
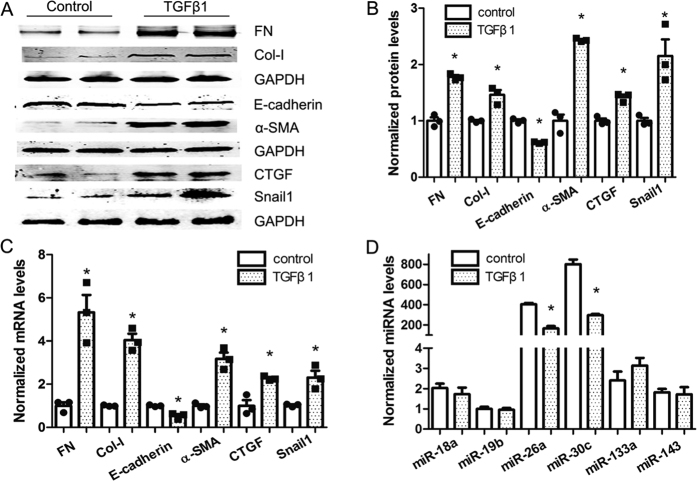
TGFβ1 induces pro-fibrotic changes and reduces miR-26a and miR-30c levels in NRK-52E cells. (**A**–**C**) TGFβ1 significantly elevated FN and Col-I, α-SMA, CTGF, and Snail1 expression but reduced E-cadherin expression as measured by (**A**) western blot, with relative expression shown in (**B**) and by (**C**) qRT-PCR. (**D**,**E**) The expression of CTGF-related miRNAs in NRK-52E cells was significantly lower 72 h after TGFβ1 treatment; U6 snRNA was measured for normalization. The bars represent the mean ± SEM (n = 3). *p < 0.05 versus control.

First, fibrotic markers were analyzed by employing qRT-PCR and western blotting in NRK-52E cells exposed to TGFβ1 (10 ng/ml). Compared with control treatment, TGFβ1 treatment for 72 h significantly augmented the expression of the myofibroblast markers fibronectin (FN), collagen type I (Col-I) and α smooth muscle actin (α-SMA) and the pro-fibrotic factors CTGF and Snail1 but reduced the expression of E-cadherin, an epithelial marker (Fig. 1A–C). These data demonstrate that TGFβ1 has the capability to induce EMT in NRK-52E cells.

We then sought to identify miRNAs that were differentially regulated in TGFβ1- treated (diabetic nephropathy condition) NRK-52E cells. An examination of several CTGF-related miRNAs revealed that miR-26a and miR-30c expression levels were the highest. Furthermore, miR-26a and miR-30c were consistently down-regulated compared with control conditions (Fig. 1D).

### miR-26a and miR-30c directly co-target CTGF, and miR-30c targets Snail1

**Figure 2:**
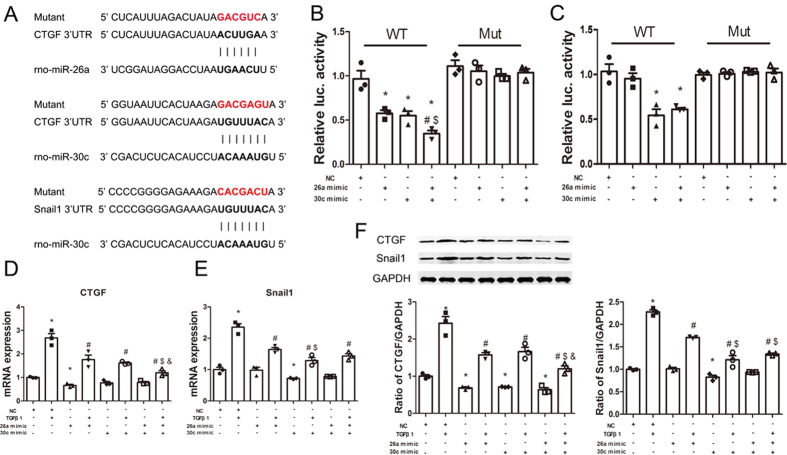
CTGF is targeted by miR-26a and miR-30c, and Snail1 is targeted by miR-30c. (**A**) Sequences of the CTGF 3′-UTR containing the miR-26a and miR-30c binding sites and the Snail1 3′-UTR containing the miR-30c binding site. The mutant sequences were used in the mutant 3′-UTR constructs. (**B**,**C**) NRK-52E cells were co-transfected with CTGF or Snail1 3′-UTR luciferase reporter plasmids (1 μg), β-galactosidase plasmid (0.2 μg), and miR-26a or miR-30c mimic alone (50 nM) or in combination (25 nM each) following the various indicated treatments. The luciferase and β-gal activities were detected using a luciferase assay kit after 72 h. The bars represent the mean ± SEM (n = 3). *p < 0.05 versus NC; ^#^p < 0.05 versus miR-26a mimic; ^$^p < 0.05 versus miR-30c mimic. (**D**,**E**) qRT-PCR analysis and (**F**) western blots of CTGF and Snail1 in NRK-52E cells transfected with miR-26a and miR-30c mimics for 72 h. The bars represent the mean ± SEM (n = 3). *p < 0.05 versus NC; ^#^p < 0.05 versus TGFβ1; ^$^p < 0.05 versus miR-26a mimic+TGFβ1; ^&^p < 0.05 versus miR-30c mimic+TGFβ1.

The computational program TargetScan predicted that the 3′-UTR of CTGF harbors two predicted binding sites for miR-26a and miR-30c. In addition, the 3′-UTR of Snail1 has one predicted binding site for miR-30c (Fig. 2A, Supplementary Figs 1 and 2). Luciferase reporter plasmids were generated to assess the direct effects of miR-26a and miR-30c on their putative target sites in the 3′-UTRs of CTGF and Snail1 mRNA. First, the CTGF 3′-UTR containing the two putative miR-26a and miR-30c target sites and the Snail1 3′-UTR containing the putative miR-30c target site were cloned into the pMIR-REPORT plasmid.

Then, cells were co-transfected with the synthetic CTGF 3′-UTR plasmid, control plasmid (β-gal), and miR-26a or miR-30c mimic alone, a combination of miR-26a/30c mimics (each at half the dose for the single mimic treatment) or negative control mimic. Consistent with our expectations, luciferase activity was significantly decreased when cells were treated with either miR-26a or miR-30c mimic alone. Interestingly, this trend was even more dramatic with the combination of miR-26a and miR-30c mimics (Fig. 2B). In contrast, no repression was observed for the construct containing the CTGF 3′-UTR with a mutated miR-26a/30c binding site (Fig. 2B).

Cells were also co-transfected with the synthetic Snail1 3′-UTR plasmid, control plasmid (β-gal), and miR-26a or miR-30c mimic alone, a combination of miR-26a/30c mimics (each at half the dose for the single mimic treatment) or negative control mimic. Luciferase activity was significantly decreased when cells were transfected with the miR-30c mimic alone but did not change when the cells were transfected with the miR-26a mimic. Luciferase activity was also significantly decreased by the combination of the miR-26a and miR-30c mimics (Fig. 2B). However, there was no difference between the miR-30c mimic group and the combination miR-26a/miR-30c mimics group. Meanwhile, no repression was observed with the construct containing the Snail1 3′-UTR with a mutated miR-30c binding site (Fig. 2B). These data demonstrate that CTGF is a genuine target of miR-26a/30c and that miR-30c also directly targets Snail1.

In addition, we explored whether miR-26a and miR-30c regulate fibrosis by targeting CTGF and Snail1. When transfected with the miR-26a or miR-30c mimic, the cellular miR-26a and miR-30c expression levels increased approximately 50- to 80-fold (Supplementary Fig. 3A,B). In the presence or absence of TGFβ1, CTGF mRNA and protein levels were markedly reduced after treatment with either miR-26a or miR-30c mimic alone; treatment with both mimics strengthened the suppressive effects on CTGF compared with treatment with either miR-26a or miR-30c mimic alone (Fig. 2D,F). Furthermore, miR-30c mimic transfection significantly decreased Snail1 mRNA (Fig. 2E) and protein expression (Fig. 2F) compared to NC transfection with or without TGFβ1 treatment. However, treatment with a combination of miR-26a and miR-30c mimics decreased Snail1 mRNA and protein expression to the same level as miR-26a or miR-30c alone, indicating that no synergy exists between these two miRNAs in suppressing Snail1 expression. Interestingly, miR-26a mimic+TGFβ1 also decreased Snail1 mRNA and protein expression, even though Snail1 3′-UTR does not contain a miR-26a target site; however, the inhibitory effect was significantly less than that of miR-30c mimic+TGFβ1. We speculate that miR-26a may indirectly regulate Snail1 via HMGA2.

### miR-26a and miR-30c mimics synergistically suppress TGFβ1-induced EMT

We investigated the synergistic effect of miR-26a and miR-30c overexpression in NRK-52E cells with or without TGFβ1 treatment. In the absence of TGFβ1, miR-26a mimic transfection into NRK-52E cells showed a trend of ameliorating EMT marker expression at both the mRNA and protein levels, but this effect was not significant compared to the NC group. In addition, miR-30c mimic transfection into NRK-52E cells significantly suppressed FN and Col-I expression at the protein level but not at the mRNA level compared to the NC group and trended towards improving EMT marker (α-SMA) expression at the mRNA and protein levels but did not reach significance. Notably, compared to the NC group, transfection with the miR-26a/miR-30c mimics combination markedly decreased FN, Col-I and α-SMA expression, but not E-cadherin expression, at the protein level. Hence, mimic-induced promotion of miR-26a and miR-30c levels may not drastically affect basal mRNA or protein levels.

**Figure 3:**
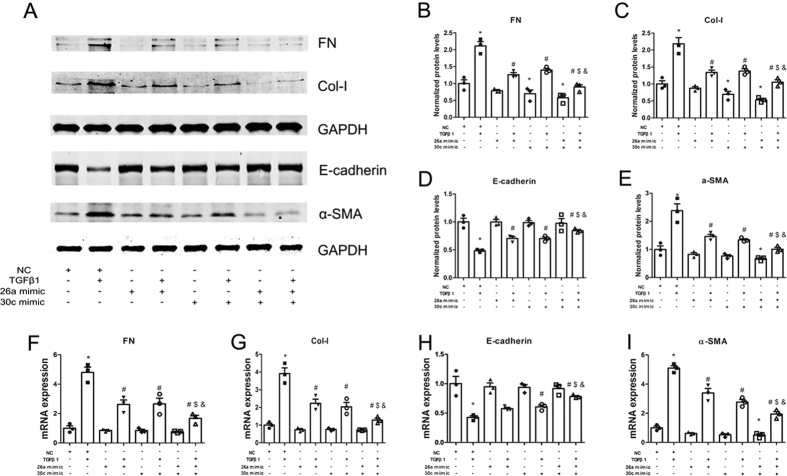
Co-treatment with the miR-26a and miR-30c mimics suppresses TGFβ1-induced EMT. (**A**) Western blot analyses of FN, Col-I, α-SMA and E-cadherin in NRK-52E cells transfected with miR control, miR-26a mimic, miR-30c mimic or a combination of the miR-26a and miR-30c mimics (each at half the dose). The results showed a significant reduction in FN, Col-I, and α-SMA and an increase in E-cadherin at the protein level. (**B**) Relative protein abundance of FN, Col-I, α-SMA and E-cadherin. (**C**) qRT-PCR. The bars represent the mean ± SEM (n = 3). *p < 0.05 versus NC; ^#^p < 0.05 versus TGFβ1; ^$^p < 0.05 versus miR-26a mimic+TGFβ1; ^&^p < 0.05 versus miR-30c mimic+TGFβ1.

Notably, in the presence of TGFβ1 and either the miR-26a or miR-30c mimic, the expression of FN, Col-I and α-SMA was markedly reduced, but E-cadherin expression was markedly increased compared with the NC+TGFβ1 group; the miR-26a and miR-30c mimics had a similar inhibitory effect on EMT. Moreover, co-transfection of NRK-52E cells with the miR-26a/miR-30c mimic combination significantly inhibited EMT compared with transfection with either mimic alone, as determined by mRNA and protein levels (Fig. 3A–I). These data show that the miR-26a and miR-30c mimics synergistically ameliorate TGFβ1-stimulated EMT in NRK-52E cells.

### miR-26a and miR-30c inhibitors collaboratively enhance TGFβ1-induced EMT

We performed a loss-of-function study by knocking down miR-26a and miR-30c alone or in combination using miRNA inhibitors. The miR-26a/30c inhibitors efficiently suppressed their expression by approximately 90% compared with the scrambled control (Supplementary Fig. 3C,D). In the absence of TGFβ1, transfection of NRK-52E cells with the miR-26a inhibitor decreased E-cadherin expression at the mRNA level but not at the protein level compared with the NC group; NRK-52E cells transfected with the miR-26a mimic showed a trend towards enhanced EMT marker expression at the mRNA and protein levels, but this change was not significant. Transfecting NRK-52E cells with the miR-30c inhibitor significantly suppressed E-cadherin expression and enhanced Col-I expression at the protein level but not at the mRNA level; FN and α-SMA expression did not significantly change. Transfection with both miR-26a and miR-30c inhibitors (each at half the dose) markedly increased Col-I and α-SMA expression but reduced E-cadherin expression at the protein level; FN expression was also increased, but this effect was not significant. Hence, inhibitor-induced repression of miR-26a and miR-30c levels may not drastically affect basal mRNA or protein levels.

**Figure 4:**
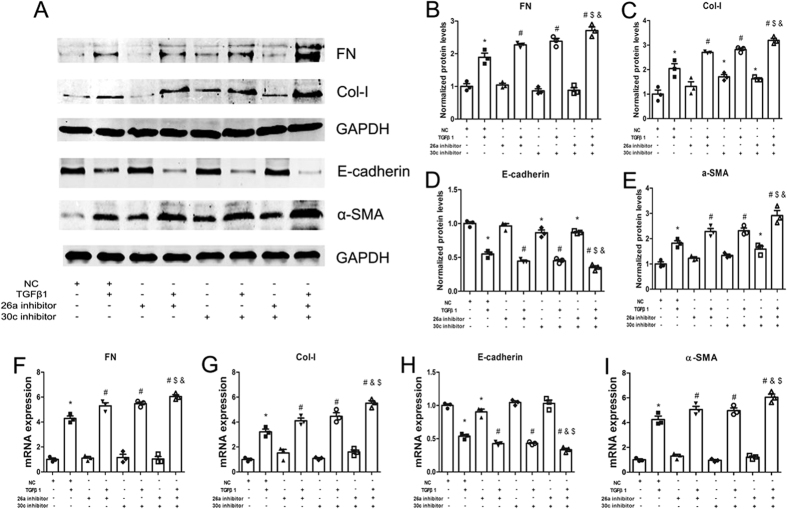
Co-treatment with the miR-26a and miR-30c inhibitors enhances TGFβ1-induced EMT. (**A**) Western blot analyses of FN, Col-I, E-cadherin and α-SMA in NRK-52E cells transfected with antimiR control, antimiR-26a, antimiR-30c or a combination of antimiR-26a and antimiR-30c (each at half the dose). The results showed significant increases in FN, Col-I, and α-SMA and a reduction in E-cadherin protein expression. (**B**) Relative protein abundance of FN, Col-I, α-SMA and E-cadherin. (**C**) qRT-PCR. The bars represent the mean ± SEM (n = 3). The data are presented as the mean ± SEM (n = 3). *p < 0.05 versus NC; ^#^p < 0.05 versus TGFβ1; ^$^p < 0.05 versus miR-26a inhibitor + TGFβ1; ^&^p < 0.05 versus miR-30c inhibitor + TGFβ1.

Notably, the TGFβ1-mediated up-regulation of fibrotic marker genes and proteins was enhanced by the individual miR-26a and miR-30c inhibitors compared with the NC+TGFβ1 group; furthermore, co-inhibition with the miR-26a and miR-30c inhibitors showed a stronger pro-fibrotic effect (Fig. 4A–I). These data show that the miR-26a and miR-30c inhibitors coordinately increase TGFβ1-induced EMT in NRK-52E cells.

### miR-26a and miR-30c regulate TGFβ1-induced EMT via CTGF/Snail1

Next, we examined whether CTGF is necessary for the coordinated regulation of EMT by miR-26a and miR-30c.

First, we compared the silencing abilities of multiple siCTGF and siSnail1 sequences in NRK-52E cells. The results showed that siCTGF-1 and siSnail1-1 had the strongest inhibitory effects, regardless of the presence of TGFβ1 (Fig. S4A,B).

NRK-52E cells were co-transfected with a combination of miR-26a/30c inhibitors and siCTGF. The up-regulation of the fibrotic marker genes FN, Col-I and α-SMA by miR-26a/30c inhibitors was diminished by siCTGF, regardless of the presence of TGFβ1 (Fig. S4C–F). E-cadherin showed the opposite trend.

We also examined whether Snail1 is necessary for the regulation of EMT by miR-30c. NRK-52E cells were co-transfected with the miR-30c inhibitor and siSnail1. The miR-30c inhibitor-mediated induction of the fibrotic marker genes FN, Col-I and α-SMA was restrained by siSnail1, regardless of the presence of TGFβ1 (Fig. S4G–J). E-cadherin showed the opposite trend.

### The potential mechanism by which miR-26a and miR-30c synergistically suppress TGFβ1-induced EMT

Next, we investigated the potential mechanism of the miR-26a and miR-30c co-regulation of TGFβ1-induced EMT in NRK-52E cells. Previous work by our group and others has shown that the activation of ERK1/2 and p38 MAPK plays important roles in the pathogenesis of DN^32^ and other diabetes-related diseases^33^,^34^. Additionally, recent studies have proposed that CTGF activates the ERK1/2^35^ and p38 MAPK^36^ signaling pathways. Therefore, we hypothesized that miR-26a and miR-30c synergistically inhibit CTGF and then restrain phosphorylation activity within the ERK1/2 and p38 MAPK signaling pathways.

**Figure 5:**
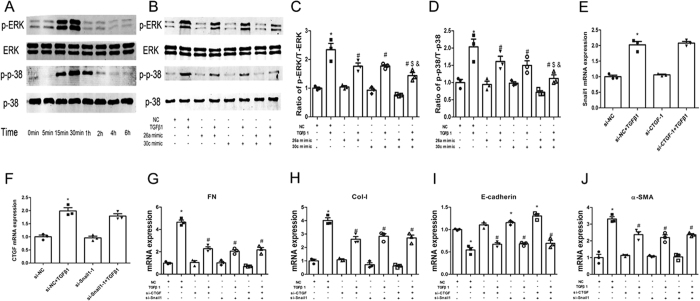
The potential mechanism by which miR-26a and miR-30c synergistically suppress TGFβ1-induced EMT. (**A**) TGFβ1 activates the ERK1/2 and p38 MAPK pathways with a peak at 30 min in NRK-52E cells. (**B**) Western blot showing the levels of total and phosphorylated ERK1/2 and p38 protein in NRK-52E cells transfected with miR control, miR-26a, miR-30c, or a combination of miR-26a and miR-30c (each at half the dose). (**C**,**D**) Relative protein abundance of total and phosphorylated ERK1/2 and p38. The data are presented as the mean ± SEM (n = 3). *p < 0.05 versus NC; ^#^p < 0.05 versus TGFβ1; ^$^p < 0.05 versus miR-26a mimic+TGFβ1; ^&^p < 0.05 versus miR-30c mimic+TGFβ1. (**E**,**F**) Gene expression analysis shows that silencing CTGF or Snail1 individually did not affect the expression of the other gene. (**G**–**J**) Gene expression analysis of FN, Col-I, E-cadherin, α-SMA in NRK-52E cells transfected with si control, siCTGF, siSnail1 or a combination of siCTGF and siSnail1 (each at half the dose). The results shows that co-treatment with siCTGF and siSnail1 did not markedly inhibit EMT. The data are presented as the mean ± SEM (n = 3). *p < 0.05 versus NC; ^#^p < 0.05 versus TGFβ1.

TGFβ1 treatment significantly increased ERK1/2 phosphorylation in NRK-52E cells compared with vehicle treatment, beginning at 15 min and peaking at 30 min, with phosphorylation levels returning to normal at 1 h. In addition, p38 MAPK phosphorylation was evident at 15 min, peaked at 30 min, and returned to normal at 2 h (Fig. 5A).

Furthermore, in the absence of TGFβ1, ERK1/2 and p38 MAPK phosphorylation was not markedly reduced by the miR-26a or miR-30c mimic compared with the NC group (0 min). Co-treatment with the miR-26a/miR-30c mimic combination showed a trend towards ameliorating ERK1/2 and p38 MAPK phosphorylation, but this effect was not significant. Hence, the mimic-induced promotion of miR-26a and miR-30c levels may not drastically affect basal ERK1/2 and p38 MAPK phosphorylation levels.

As expected, activation of the ERK1/2 and p38 MAPK signaling pathways due to TGFβ1 treatment (30 min) was markedly attenuated by the individual miR-26a and miR-30c mimics. Furthermore, co-treatment with the miR-26a/miR-30c mimics further enhanced the inhibitory effect (Fig. 5B–D). Hence, miR-26a and miR-30c effectively and synergistically inhibited the TGFβ1-induced activation of MAPKs in TGFβ1-treated NRK-52E cells.

CTGF and Snail1 are important transcription factors that trigger EMT in DN. Previous studies reported that silencing CTGF^7^,^37^ and Snail1^38^ inhibits EMT and fibrosis in the kidney. Thus, determining whether CTGF and Snail1 collaboratively suppress TGFβ1-induced EMT was also important for explaining the potential mechanism of miR-26a/30c co-regulation.

Interestingly, gene expression analysis showed that silencing CTGF did not affect Snail1 mRNA levels and that silencing Snail1 did not affect CTGF mRNA levels (Fig. 5C,D).

In the absence of TGFβ1, silencing CTGF or Snail1 alone did not drastically affect basal EMT marker mRNA expression compared with the NC group. In the presence of TGFβ1, silencing CTGF or Snail1 individually markedly suppressed TGFβ1-induced EMT compared with the NC+TGFβ1 group, as determined by mRNA levels. However, compared with the individual treatments, co-treatment with both siCTGF and siSnail1 (each at half the dose) did not markedly inhibit EMT at the mRNA level (Fig. 5I–L). These data suggest that CTGF and Snail1 are independent transcription factors mediating EMT in NRK-52E cells.

### miR-26a and miR-30c are down-regulated in the renal cortices of diabetic OLETF rats

**Figure 6:**
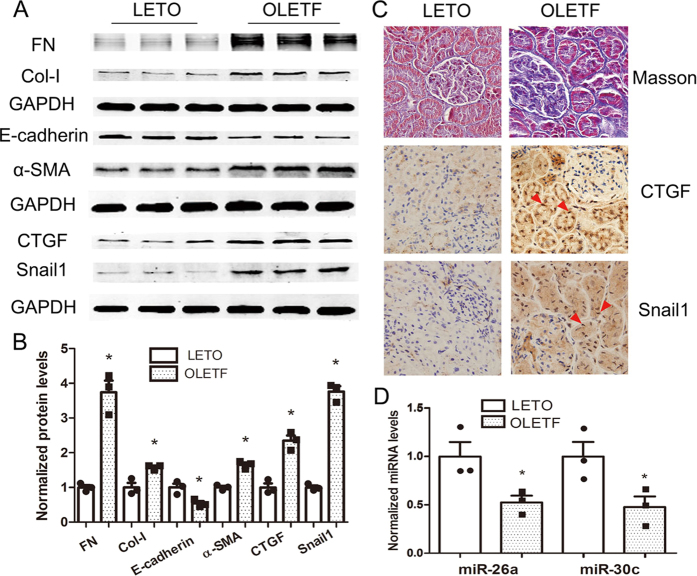
Changes in miR-26a and miR-30c expression in the renal cortices of 40-week-old diabetic OLETF rats. (**A**) Western blot showing that FN, Col-I, E-cadherin, α-SMA, CTGF and Snail1 expression levels were increased in OLETF rats. (**B**) Relative abundance of FN, Col-I, E-cadherin, α-SMA, CTGF and Snail1 in LETO and OLETF rats. (**C**) Masson’s trichrome stain analysis of renal cortices from LETO and OLETF rats showing extensive collagen deposition in the tubular interstitial area (magnification, ×400). Immunohistochemistry showing that CTGF and Snail1 expression was significantly increased in tubular epithelial cells and renal interstitial areas in OLETF rats compared with LETO rats (magnification, ×400). Moreover, CTGF and Snail1 expression was also concentrated in renal tubular epithelial cell nuclei (arrows). (**D**) The relative expression of miR-26a and miR-30c extracted from the renal cortex was significantly lower in 40-week-old diabetic OLETF rats. U6 snRNA was measured for normalization. Data are presented as the mean ± SEM (n = 3). *p < 0.05 versus LETO.

To explore the *in vivo* correlation between miR-26a/30c and DN, we compared the expression of CTGF/Snail1 and miR-26a/30c in the renal cortices of 40-week-old OLETF rats (diabetic) and LETO rats (non-diabetic). Significant increases in the levels of the myofibroblast markers FN, Col-I and α-SMA and the pro-fibrotic factors CTGF and Snail1 were also observed in the OLETF rats (Fig. 6A,B). Masson staining revealed more extensive tubular interstitial collagen deposition in the OLETF group, which confirmed that the 40-week-old OLETF rats exhibited tubulointerstitial fibrosis (Fig. 6C). Immunohistochemistry showed that CTGF and Snail1 expression was significantly increased in renal tubules and interstitial areas in OLETF rats compared with LETO rats. CTGF and Snail1 expression was also higher in renal tubular epithelial cell nuclei in the OLETF rats (Fig. 6C). These changes paralleled the decreases in the expression of miR-26a and miR-30c in the kidney cortex of OLETF rats compared with non-diabetic LETO rat controls (Fig. 6D). Taken together, these results demonstrate that the signaling cascade established *in vitro* was also verified in an *in vivo* rat model of DN.

### miR-26a is up-regulated in urinary extracellular vesicles of DN patients

miRNAs play an important role in diagnostics. There have been no previous reports on the expression of miR-26a/30c in urinary extracellular vesicles in DN. A recent report indicated that miR-26a and miR-30c were among the top 10 highly expressed miRNAs in urinary extracellular vesicles^39^. We explored the concentrations of miR-26a/30c in urinary extracellular vesicles and assessed the potential of urinary extracellular vesicle miR-26a/30c levels as a DN marker.

First-morning urine samples were obtained from 50 patients with DM and DN. The demographic and clinical parameters of the DM and DN patients are shown in Supplementary Table 1. Urinary extracellular vesicles were characterized by a round bilayer lipid membrane structure with a size between 20 and 147 nm using an electron microscope (Supplementary Fig. 5A).

miR-26a levels were higher in DN patients than in DM patients (Table 1). However, there was no significant difference in miR-30c levels between DN and DM patients (Table 1). This result indicated that urinary extracellular vesicle miR-26a levels might be a new marker for DN. Further analysis showed that there was no significant correlation between cystatin, urinary albumin excretion rate (UAER), serum creatinine (Scr), estimated glomerular filtration rate (eGFR) or HbA1C% and the levels of miR-26a and miR-30c. The correlation between miR-26a levels and UAER was close to reaching significance (p = 0.057); perhaps the limited number of patients hindered this analysis (Supplementary Table 2).

To explore whether TGFβ1 influences miR-26a and miR-30c expression in extracellular vesicles, we isolated this compartment from NRK-52E cell supernatants. Extracellular vesicles in cell culture medium, which have a similar structure to urinary extracellular vesicles, were also identified by electron microscopy (Supplementary Fig. 5B). miR-26a expression was significantly increased by four-fold in extracellular vesicles after TGFβ1 treatment, but miR-30c expression did not change (Supplementary Fig. 6).

## Discussion

miRNAs are key players in the development of DN. The results presented in this study indicate that miR-26a, which targets CTGF, and miR-30c, which targets CTGF and Snail1, provide significant renal protection in renal tubular epithelial cells. Furthermore, the combination of both miRNAs leads to additive efficacy in decreasing the occurrence of EMT, suggesting that multi-target therapy using different classes of miRNAs is a potential strategy for the future treatment of DN.

The role of miR-26a in DN remains debatable. In one study, enhanced miR-26a expression induced mesangial cell hypertrophy and increased matrix protein expression^40^. However, in another study, miR-26a inhibited TGFβ1-induced ECM protein expression in podocytes^25^. Both mesangial cells and podocytes are present in glomeruli. However, the function of miR-26a has not been previously explored in renal tubular epithelial cells, another important component of the kidney. Here, we showed that miR-26a plays a protective role by down-regulating CTGF in renal tubular epithelial cells. Interestingly, miR-26a also down-regulated Snail1 expression; however, miR-26a did not directly down-regulate Snail1 luciferase activity, suggesting that Snail1 is not a direct target of miR-26a. We hypothesize that miR-26a influences other signaling pathways to decrease Snail1 expression. One potential mechanism is that miR-26a directly targets HMGA2^41^, which directly binds to the Snail1 promoter^42^. Further detailed studies are necessary to elucidate the exact mechanisms. Interestingly, TGFβ1 treatment down-regulated the expression of CTDSP2 and CTDSPL, which are the miR-26a host genes, and of NF-YC, which is the miR-30c host gene. These findings could further explain how TGFβ1 directly down-regulates miR-26a and miR-30c.

Although a single gene can elicit an effect or phenotypic disturbance, multiple miRNAs targeting a common gene may be more efficient. The cooperative roles of miRNAs are important for the treatment of DN because they might enable the use of lower doses of individual miRNAs and thus increase treatment selectivity^43^. The coordinated roles of miR-26a and miR-30c have not been investigated previously. Bioinformatic analyses revealed that the miR-26a and miR-30c target sites in the 3′-UTR of CTGF are non-overlapping (Supplementary Fig. 1). Previous studies showed that synergistic effects occur with different types of miRNAs, e.g., miRNAs that belong to a single cluster but do not share homology^30^, two mature products of one miRNA^44^ or two independent miRNAs^29^,^31^. The mechanisms underlying the coordinated roles of miRNAs are complex and require more detailed study.

The mechanisms underlying the coordinated roles of miR-26a and miR-30c may stem from the cooperation between the CTGF and Snail1 transcription factors. Previous studies have shown that CTGF and Snail influence each other. Snail expression significantly increased in human lens epithelial cells after CTGF treatment^45^, and Snail induced CTGF expression in MCF-7 breast cancer cells^46^. However, the relationship between CTGF and Snail has not been examined in DN. Here, we showed that silencing CTGF or Snail1 did not influence the expression of the other gene and that co-silencing both targets did not synergistically suppress TGFβ1-induced EMT in NRK-52E cells, implying that CTGF and Snail1 are independent transcription factors that cannot explain the coordinated roles of miR-26a and miR-30c.

MAPK signaling is a well-known modulator in renal EMT. Moreover, Elsa *et al*. showed that CTGF could inhibit the MAPK signaling pathway in proximal tubular epithelial cells^36^. In our study, miR-26a and miR-30c synergistically suppressed CTGF and further inhibited the ERK1/2 and p38 MAPK signaling pathways. This result may partly explain the coordinated roles of miR-26a and miR-30c in DN. Nevertheless, we could not confirm whether other signaling pathways mediate the renoprotective property of these miRNAs. The coordinated roles of miR-26a and miR-30c are complicated and require detailed study.

To further explore the relevance of our findings *in vivo*, we investigated CTGF, Snail1 and miR-26a/30c expression in the renal cortices of 40-week-old OLETF rats. Similar to our *in vitro* findings, miR-26a/30c expression was down-regulated in the renal cortices of OLETF rats compared with LETO rats. The results further confirmed that CTGF, Snail1 and miR-26a/30c play important roles in DN.

Recently, an increasing number of studies have shown that urinary extracellular vesicles are diagnostic markers in DN and other kidney diseases. In addition, the diagnosis of DN is still controversial. Herein, we wanted to determine whether miRNA in extracellular vesicles can distinguish DN from DM patients. Here, we showed that the miR-26a level was significantly increased in DN patients compared with DM patients but that the miR-30c level was not different. Urinary extracellular vesicle miR-26a expression may be associated with DN progression, and miR-26a may become a new DN diagnostic marker. Interestingly, miR-26a expression was up-regulated in urinary extracellular vesicles from DN patients but was decreased in TGFβ1 treated renal tubular epithelial cells and the kidney cortex of OLETF rats. A subsequent experiment found that the miR-26a level was significantly increased in extracellular vesicles from NRK-52E cell supernatants after TGFβ1 treatment. Because the origin of miR-26a in urinary extracellular vesicles is complex, we could not verify whether miR-26a secreted by renal tubular epithelial cells was the primary contributor to the increased presence in urinary extracellular vesicles. Additional in-depth studies are necessary to elucidate the detailed mechanism. To further validate miR-26a as a new diagnostic biomarker, a larger sample size in a prospective experiment is needed.

**Figure 7:**
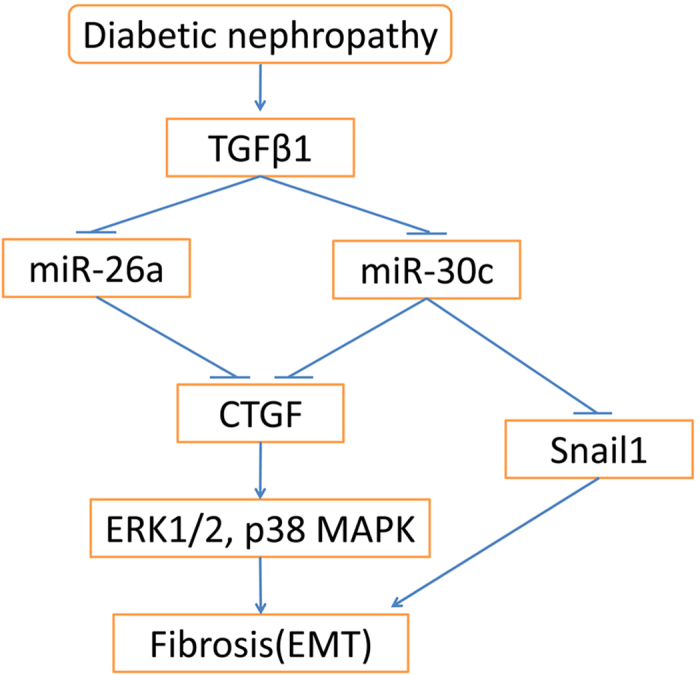
TGFβ1-miR-26a/30c signaling network leading to kidney fibrosis in diabetic nephropathy. Under diabetic nephropathy conditions, the TGFβ1 level is increased, resulting in reduced miR-26a and miR-30c expression. miR-26a and miR-30c coordinately inhibit CTGF expression, leading to decreased phosphorylation of ERK1/2 and p38. In addition, miR-30c can suppress Snail expression. ERK1/2 and p38 suppression and Snail1 expression together contribute to kidney fibrosis. This new multicomponent network contributes to the pathogenesis of diabetic nephropathy.

In summary, we present a new regulatory model in renal tubular epithelial cells (Fig. 7), in which miR-26a and miR-30c form an miRNA network that synergistically modulates the TGFβ1-mediated fibrotic response via coordinated inhibition of CTGF-dependent pathways and further inhibits the ERK1/2 and p38 MAPK signaling pathways. Taken together, these results describing the coordination of miR-26a/30c in renal fibrosis provide new insights into the progression of DN, and co-silencing miR-26a and miR-30c could be a novel renoprotective therapy for DN patients.

## Materials and Methods

### Cell culture

NRK-52E cells (ATCC, Rockville, MD, USA) were grown in DMEM (Gibco, China) containing 4.5 g/l glucose and 10% FBS (Sijiqing, China). Cells were cultured in a 5% CO_2_ incubator at 37 °C until 70–80% confluence. Cells were treated with 10 ng/ml human recombinant TGFβ1 (Invitrogen, CA, USA)^47^,^48^.

### RNA extraction and qRT-PCR

Total RNA was extracted from NRK-52E cells and rat kidney cortices with TRIzol (DingGuo, China). Reverse transcription of total RNA was performed with an M-MLV kit (Invitrogen, CA, USA). The gene expression levels of FN, Col-I, E-cadherin, α-SMA, CTGF, Snail1 and β-actin were determined using a Roche LightCycler 480 Real-Time PCR System (Roche, Switzerland). The relative values for each gene were calculated by the comparative 2^−ΔΔCt^ method with β-actin as an internal control. The primer sequences (Invitrogen, Shanghai, China) were as follows: FN: 5′-TGGAGAGACAGGAGGAAATAGC-3′ and 5′-CAGTGACAGCATACAGGGTGAT-3′; Col-I: 5′-ACATGCCGTGACCTCAAGAT-3′ and 5′-ATGTCCATTCCGAATTCCTG-3′; E-cadherin: 5′-CACCGTGGTTTCTTGCGTTT-3′ and 5′-TCAGGTTCACTGGCATGCTT-3′; α-SMA: 5′-TGGATCAGCGCCTTCAGTTC-3′ and 5′-GGCCAGGGCTAGAAGGGTA-3′; CTGF: 5′-TGGCTTGCTCAGGGTAACTG-3′ and 5′-AACTGCCTCCCAAACCAGTC-3′; Snail1: 5′-CGGAAGCCCAACTATAGCGA-3′ and 5′-AGAGTCCCAGATGAGGGTGG-3′; and β-actin: 5′-GCGAGTACAACCTTCTTGCAG-3′ and 5′-GCCTTGCACATGCCGGA-3′.

### miRNA isolation and qRT-PCR

Previously obtained total RNA was reverse transcribed using an miRNA First-Strand cDNA Synthesis Kit (TianGen, China) in accordance with the manufacturer’s instructions. A miRcute miRNA Detection Kit (TianGen, China) was used for quantitative PCR on a Roche LightCycler 480 Real-Time PCR System (Roche, Switzerland). The relative values of target miRNAs were calculated by the comparative 2^−ΔΔCt^ method with U6 snRNA as an internal control. Primers for miR-26a, miR-30c, and U6 were purchased from TianGen.

### Western blotting

Equal amounts of protein (50 μg), which was extracted from NRK-52E cells and rat kidney cortices, were electrophoresed on 8–12% SDS-PAGE gels, transferred to PVDF, blocked and then incubated with the following primary antibodies at 4 °C overnight: FN (Sigma, St. Louis, MO, USA), α-SMA (Boster, China), E-cadherin (BD, USA), col-I (Merck Millipore, Germany), CTGF (Abcam, Cambridge, MA), p-ERK1/2 (Cell Signaling, MA, USA), ERK1/2 (Cell Signaling, MA, USA), p-p38 MAPK (Cell Signaling, MA, USA), p38 MAPK (Cell Signaling, MA, USA), Snail1 (Abcam, Cambridge, MA) and GAPDH (ZSGB-BIO, China). A fluorescent secondary antibody (LI-COR, USA) was then incubated with the blots at room temperature for 1 h. The images were acquired using an Odyssey infrared imaging system (LI-COR, USA). Quantity One software (Bio-Rad, USA) was used for the densitometry analysis.

### Luciferase reporter assay

The CTGF 3′-UTR-luciferase reporter plasmid was constructed using the pMIR-REPORT vector (Ambion, USA), and nucleotides 1–201 and 1068–1268 of the CTGF 3′-UTR were cloned into the plasmid. The two regions of the predicted binding sites for miR-26a and miR-30c were separated by the interval AAAAAA (Sangon Biotech, Shanghai, China). Mutated plasmids were constructed to contain two mutated seed sequences for miR-26a and miR-30c (from ACTTGA to GACGTC for the miR-26a binding site and from TGTTTAC to GACGAGT for the miR-30c binding site). Similarly, the Snail1 3′-UTR-luciferase reporter plasmid contained nucleotides 516–731 of the Snail1 3′-UTR sequence. The mutated plasmid contained a mutated miR-30c seed sequence (from TGTTTAC to CACGACT). pMIR-REPORT β-gal, a beta-galactosidase (β-gal) reporter plasmid (Ambion, USA), was used as a transfection control.

For the luciferase reporter assays, NRK-52E cells were seeded in 24-well plates and transfected with 1 μg of pMIR-REPORT plasmid, 0.2 μg of β-gal plasmid, and 50 nmol miR-26a or miR-30c mimic, 25 nmol miR-26a and 25 nmol miR-30c mimic, or 50 nmol scrambled negative control RNA using Lipofectamine^®^ 3000 (Invitrogen, CA, USA) in OptiMEM (Gibco, CA, USA) with β-gal as a transfection control^47^. The cells were harvested 72 h after transfection and analyzed using luciferase assay kits (Beyotime, China). All experiments were performed in triplicate.

### miRNA mimic/inhibitor and siRNA transfection

For the miRNA overexpression experiment, 50 nM miR-26a mimic, 50 nM miR-30c mimic or 25 nM each of miR-26a and miR-30c mimic (RiboBio, Guangzhou, China) was used. For the miRNA silencing experiment, 150 nM miR-26a inhibitor, 150 nM miR-30c inhibitor or 75 nM each of miR-26a and miR-30c inhibitor (RiboBio, Guangzhou, China) was used. For the gene knockdown experiment, 50 nM CTGF siRNA, 50 nM Snail1 siRNA or 25 nM each of CTGF and Snail1 siRNA (RiboBio, Guangzhou, China) was used. NRK-52E cells were transfected using Lipofectamine^®^ 3000 according to the manufacturer’s instructions. The cells were harvested 72 h after transfection for RNA and protein analysis.

To ascertain the impact of miR-26a and miR-30c on the ERK1/2 and p38 MAPK signaling pathways, 50 nM miR-26a mimic, 50 nM miR-30c mimic or 25 nM each of miR-26a and miR-30c mimic (RiboBio, Guangzhou, China) was applied to the cells 30 min after TGFβ1 or control treatment.

### *In vivo* studies

The 40-week-old OLETF rats with spontaneous type 2 diabetes and healthy LETO control rats (n = 3 for each group) used in this study were described previously^49^,^50^. All of the experiments were approved by the Southern Medical University’s institutional review board, and the animal studies were performed in accordance with the Institutional Animal Care Guidelines.

### Renal histopathology

Rat kidney cortex samples were used for immunohistochemical analyses as described previously^34^,^49^. Tissue sections were stained with Masson’s trichrome stain (Maiwei, China). Tissue sections were also stained with anti-CTGF polyclonal antibody (ABclonal, USA) or anti-Snail1 polyclonal antibody (ABclonal, USA), followed by HRP-conjugated secondary antibody. Finally, slices were stained with hematoxylin (KeyGEN bioTECH, China). All images were obtained using an Olympus microscope (Japan).

### Isolation and detection of extracellular vesicle miRNAs

First-morning urine from all of the DM and DN patients (see additional information for inclusion and exclusion criteria) was collected. Extracellular vesicles from urine and cell culture medium were subjected to differential centrifugation as previously described^22^. The pellets were suspended in 100 μl of PBS and stored at −80 °C. The presence of extracellular vesicles was verified by transmission electron microscopy as previously described^22^. miRNA was isolated from extracellular vesicles using TRIzol. Notably, 25 fmol cel-miR-39 (TIANGEN, Beijing, China) was added to extracellular vesicle samples after a 5 min incubation in TRIzol as previously described^22^. miRNA expression values were normalized to those of cel-miR-39.

This study was approved by the ethics committee of Nanfang Hospital, Southern Medical University, and conducted in accordance with the guidelines of the ethical management. All of the patients provided signed informed consent prior to participating in the study.

### Statistics

All data were analyzed using SPSS 21.0. All results are presented as the mean ± SEM. A two-tailed Student’s t-test was used for comparisons of independent groups. One-way ANOVA was used to compare three or more independent groups, the least significant difference (LSD) test was used for multiple comparisons, and Dunnett’s T3 procedure was used for heterogeneous variances. Spearman rank correlation analysis was used for correlation analysis. P values less than 0.05 were considered significant.

## Additional Information

**How to cite this article**: Zheng, Z. *et al*. The coordinated roles of miR-26a and miR-30c in regulating TGFβ1-induced epithelial-to-mesenchymal transition in diabetic nephropathy. *Sci. Rep.* **6**, 37492; doi: 10.1038/srep37492 (2016).

**Publisher's note:** Springer Nature remains neutral with regard to jurisdictional claims in published maps and institutional affiliations.

## Supplementary Material

Supplementary Information

## Figures and Tables

**Table 1:** Urinary extracellular vesicle expression of miR-26a and miR-30c.

	DM (n = 30)	DN (n = 20)	*Z*	*P*
miR-26a	0.0011 ± 0.0033	0.0036 ± 0.0065	2.753	0.006
miR-30c	0.0057 ± 0.0072	0.0074 ± 0.0132	0.861	0.389

Changes in miR-26a and miR-30c expression in urinary extracellular vesicles from DN patients. Expression of miR-26a was significantly elevated in urinary extracellular vesicles from DN patients.
