# Supplementary material for: The coordinated roles of miR-26a and miR-30c in regulating TGFβ1-induced epithelial-to-mesenchymal transition in diabetic nephropathy
Source: Sci Rep. 2016 Nov 22;6:37492. doi: 10.1038/srep37492 (PMC5118685; doi:10.1038/srep37492)
Supplement: Supplementary Information [file srep37492-s1.pdf]

Supplementary information

The coordinated roles of miR-26a and miR-30c in regulating TGFβ1-induced epithelial-to-mesenchymal transition in diabetic nephropathy

Zongji Zheng*, Meiping Guan*, Yijie Jia*, Dan Wang, Ruoyu Pang, Fuping Lv, Zhizhou Xiao, Ling Wang, Hongbin Zhang and Yaoming Xue

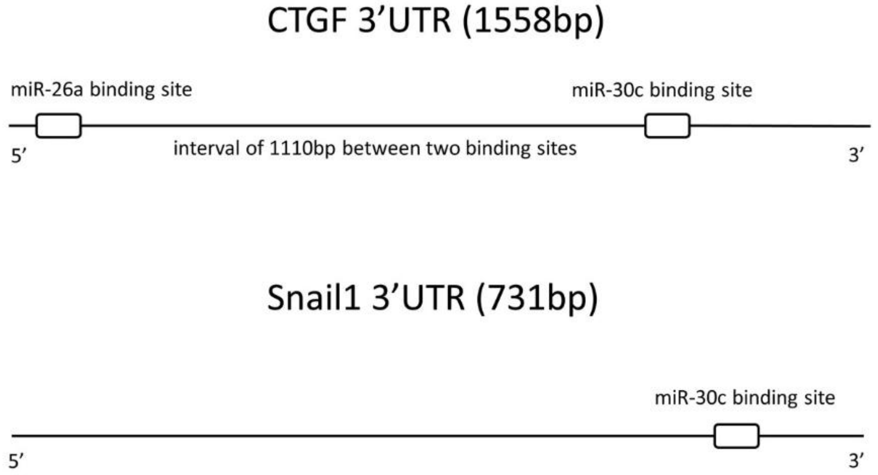

Figure 1. Schematic of the miR-26a/30c binding sites in the CTGF and Snail1 3’-UTRs.

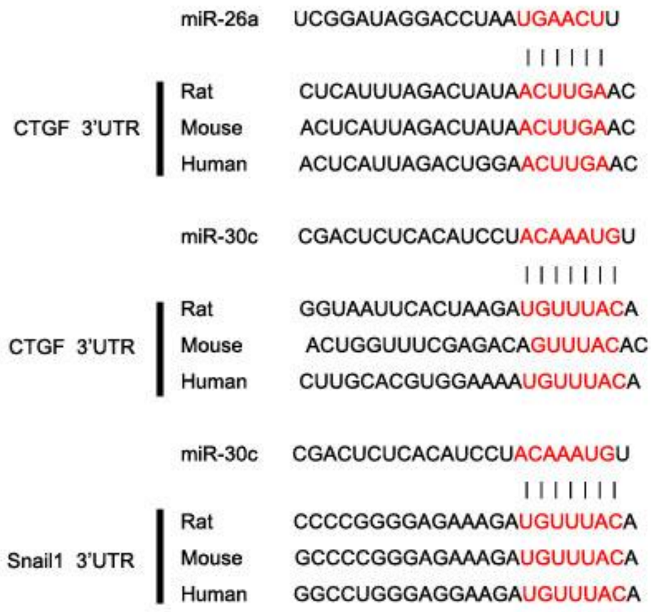

Figure 2. miR-26a and miR-30c binding sites in the human, rat and mouse CTGF and Snail1 3'-UTRs. The seed sequences are shown in red.

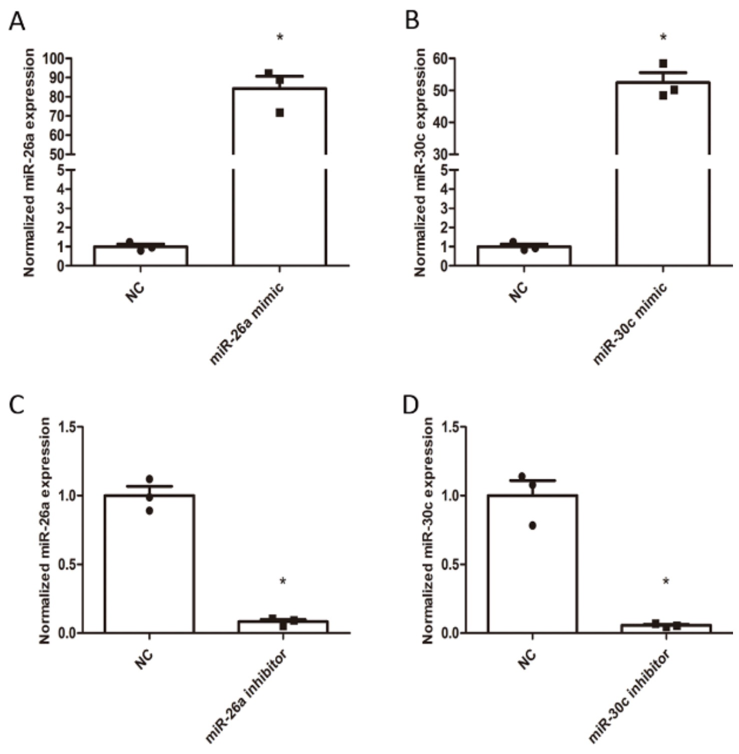

Figure 3. The effect of miR-26a/30c mimic or inhibitor on miR-26a/30c in NRK-52E cells. (A-B) miR-26a/30c mimics significantly elevated miR-26a/30c expression. (C-D) miR-26a/30c inhibitors significantly decreased miR-26a/30c expression. U6 snRNA was measured for normalization. The bars represent the mean ± SEM (n=3). *p<0.05 versus NC.

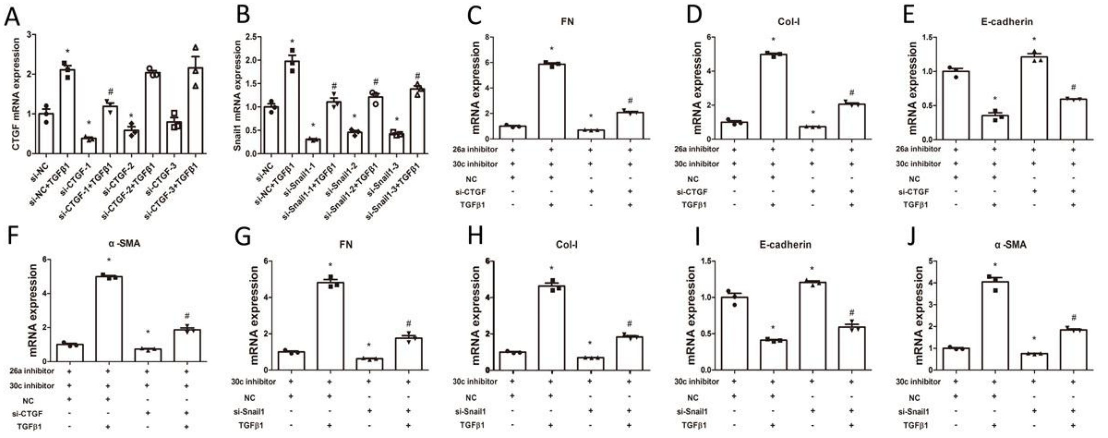

Figure 4. miR-26a and miR-30c regulate TGFβ1-induced EMT via CTGF or Snail1. (A-B) Silencing abilities of three siCTGF and siSnail1 sequences in NRK-52E cells. (C-F) siCTGF abolished the up-regulation of fibrotic marker genes by miR-26a and miR-30c inhibitors, regardless of the presence of TGFβ1. (G-J) siSnail1 alleviated the up-regulation of fibrotic marker genes by miR-30c inhibitors, regardless of the presence of TGFβ1. The data are presented as the mean ± SEM (n=3). *$p<0.05$ versus NC; #$p<0.05$ versus TGFβ1.

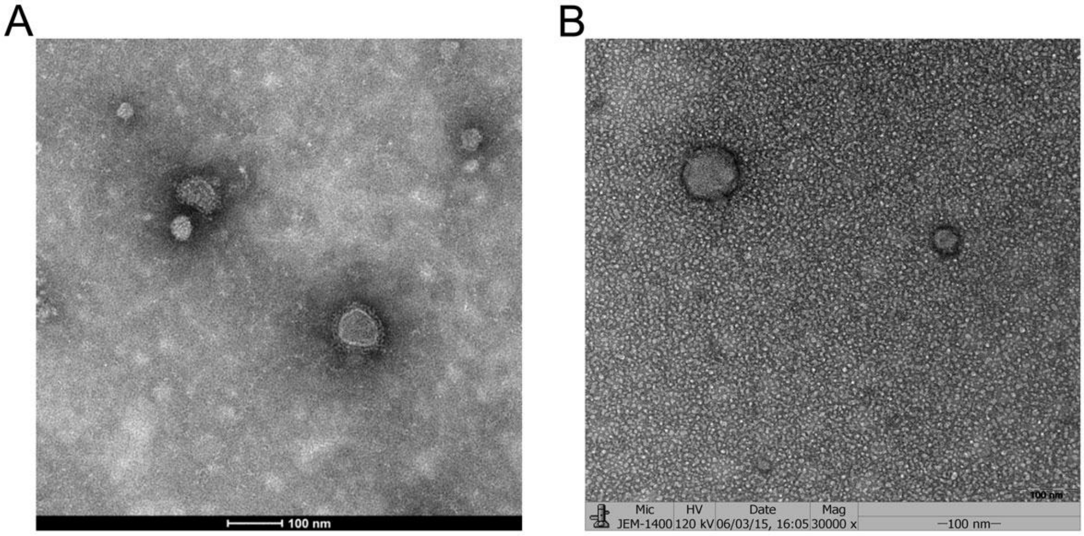

Figure 5. Transmission electron microscopy photograph of extracellular vesicles in urine (A) and cell culture medium; (B) these vesicles are characterized by a round bilayer lipid membrane structure with a size between 20 and 147 nm.

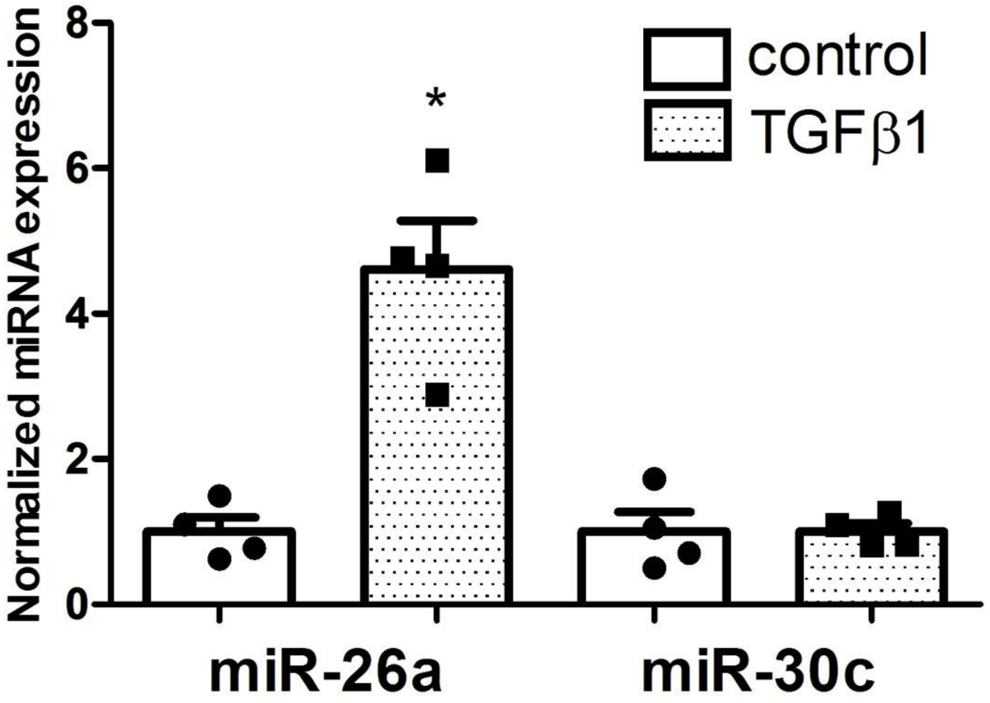

Figure 6. miR-26a and miR-30c expression in extracellular vesicles from NRK-52E cell supernatant. miR-26a expression was significantly elevated in extracellular vesicles after TGFβ1 treatment, but miR-30c expression did not change. The bars represent the mean ± SEM (n=4). *$p<0.05$ versus control.

Patient inclusion and exclusion criteria.

The inclusion and exclusion criteria were consistent with those in our previous study15. A total of 50 patients who were diagnosed with T2DM and admitted to the Department of Endocrinology & Metabolism, Nanfang Hospital, Southern Medical University, between October 2014 and May 2015 were enrolled in this study. According to the degree of albuminuria, patients were classified into the DM group (ACR<2.5 mg/mmol) or the DN group (ACR>25 mg/mmol).

The exclusion criteria were as follows: active urinary tract infection, nondiabetic kidney diseases, neoplastic disorders, severe liver disease, inflammatory disorders, pregnancy, or a recent history of acute myocardial infarction, stroke, or occlusive peripheral vascular disease. Patients with an eGFR<15 mL/min/1.73 m^2 and those with macroalbuminuria/microalbuminuria who did not present with diabetic retinopathy were excluded.

	DM (n=30)	DN (n=20)	*t*	*P*
Age (years)	57.90±8.223	55.00±8.072	1.235	0.224
Sex (male/female)	30/4	20/2	1.403	0.161
Duration of diabetes (years)	7.08±6.29	8.33±7.38	0.643	0.523
BMI	24.76±3.35	25.47±2.72	0.790	0.433
SBP (mmHg)	136.00±22.86	149.80±22.48	2.11	0.041
DBP (mmHg)	82.23±10.61	90.50±14.69	2.168	0.038
HbA1C%	9.43±2.31	9.47±1.70	0.058	0.954
Scr (μmol/L)	74.47±14.91	91.10±36.28	1.94	0.064
Cystatin (mg/L)	0.98±0.23	1.44±0.53	3.69	0.001
TC (mmol/L)	4.87±0.88	4.53±0.90	1.306	0.198
TGs (mmol/L)	1.31±0.51	2.82±1.75	3.67	0.002
HDL-C (mmol/L)	1.00±0.17	0.99±0.26	0.149	0.882
LDL-C (mmol/L)	3.12±0.90	2.75±0.74	1.512	0.137
eGFR (mL/min/1.73 m^2)	94.78±15.38	86.82±26.65	1.325	0.192
UACR (mg/mmol)	0.98±0.56	210.03±221.32	4.224	<0.001
UAER (mg/24 h)	9.44±4.06	1742.8±1659.8	4.67	<0.001

Table 1. The demographic and clinical characteristics of the recruited subjects. There were no significant differences in age, gender, duration of diabetes, BMI, HbA1C%, Scr, TC, HDL-C, LDL-C or eGFR between the two groups. The SBP, DBP, cystatin, TGs, urine albumin-to-creatinine ratio (UACR), and urinary albumin excretion rate (UAER) were higher in the DN patients.

miR-26a	Cystatin (mg/L)	UAER (mg/24 h)	Scr (μmol/L)	eGFR (mL/min/1.73 m^2)	HbA1C (%)
r	0.120	0.271	-0.042	0.112	0.034
P	0.406	0.057	0.773	0.444	0.814

miR-30c	Cystatin (mg/L)	UAER (mg/24 h)	Scr (μmol/L)	eGFR (mL/min/1.73 m^2)	HbA1C (%)
r	-0.249	-0.190	-0.208	0.252	-0.04
P	0.081	0.187	0.148	0.081	0.781

Table 2. Correlations between urinary extracellular vesicles miRNA levels and renal function parameters in DM (n=30) and DN patients (n=20). The results indicated no correlation between miR-26a or miR-30c levels and cystatin, urinary albumin excretion rate (UAER), serum creatinine (Scr), estimated glomerular filtration rate (eGFR) or HbA1C%; however, the correlation between miR-26a levels and UAER was close to reaching significance (p=0.057).
